# The Independent Effects of Kidney Length and Vascular Plaque on Ten-Year Outcomes of Extended Criteria Donor Kidney Transplants

**DOI:** 10.3389/ti.2023.11373

**Published:** 2023-07-14

**Authors:** Bekir Tanriover, Darren Stewart, Layla Kamal, Muhammad Saeed, Matthew Cooper, Julia Foutz, Harrison McGehee, Gaurav Gupta

**Affiliations:** ^1^ Division of Nephrology, University of Arizona, Tucson, AZ, United States; ^2^ Department of Surgery, New York University Langone Health, New York City, NY, United States; ^3^ Division of Nephrology, Virginia Commonwealth University, Richmond, VA, United States; ^4^ Medical College of Wisconsin, Milwaukee, WI, United States; ^5^ United Network for Organ Sharing, Richmond, VA, United States

**Keywords:** kidney anatomy, length, vascular plaque, expanded criteria donor, deceased donor

## Abstract

The independent effects of deceased donor kidney length and vascular plaque on long-term graft survival are not established. Utilizing DonorNet attachments from 4,480 expanded criteria donors (ECD) recovered between 2008 and 2012 in the United States with at least one kidney biopsied and transplanted, we analyzed the relationship between kidney length and vascular plaques and 10-year hazard of all-cause graft failure (ACGF) using causal inference methods in a Cox regression framework. The composite plaque score (range 0–4) and the presence of any plaque (yes, no) was also analyzed. Kidney length was modeled both categorically (<10, 10–12, >12 cm) as well as numerically, using a restricted cubic spline to capture nonlinearity. Effects of a novel composite plaque score 4 vs. 0 (HR 1.08; 95% CI: 0.96, 1.23) and the presence of any vascular plaque (HR 1.08; 95% CI: 0.98, 1.20) were attenuated after adjustment. Likewise, we identified a potential nonlinear relationship between kidney length and the 10-year hazard of ACGF, however the strength of the relationship was attenuated after adjusting for other donor factors. The independent effects of vascular plaque and kidney length on long-term ECD graft survival were found to be minimal and should not play a significant role in utilization.

## Graphical Abstract

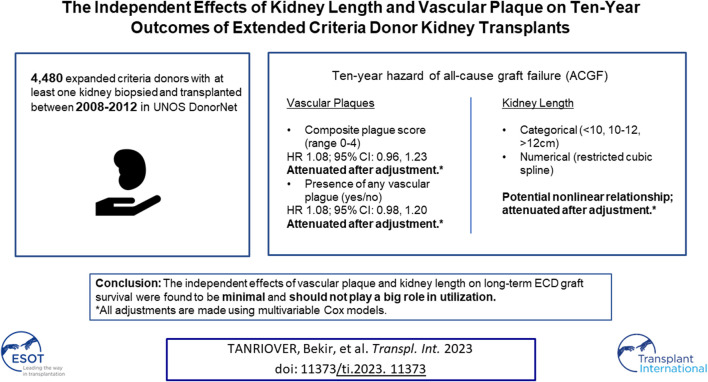

## Introduction

The assessment of deceased donor kidney anatomy (specifically regarding kidney length and vascular plaque) can influence whether kidneys are transplanted or ultimately discarded [1, 2]. Surgical evaluation can provide valuable information regarding kidney size (length and weight) [3, 4], atherosclerosis (vascular plaques affecting aorta and renal artery) [2, 5], anatomical variations (number of donor renal arteries and ureters) [6], injuries, renal tumors, infarcts, thrombosis, scarring, and *ex-vivo* organ perfusion. The surgical appraisal is particularly critical for extended criteria donors (ECD), which have comprised 20.4% of deceased donor pool with an average kidney donor profile index (KDPI) of 85% and a disproportionately high discard rate (exceeding 50%) during the past decade in the United States (U.S.) [7–10].

In initial results from our BARETO (Biopsy, Anatomy & Resistance Effects on Transplant Outcomes) study [11], we reported the independent effects of procurement biopsy findings on long-term renal graft survival in ECD transplants. In the study cohort, across four GS categories (0%–5%, 6%–10%, 11%–15%,16%–20%, 21+%), donor characteristics, as expected, included following noteworthy comorbidities: older age (the mean age range from 59.4 ± 5.8 to 60.2 ± 6.1 years), a relatively high prevalence of hypertension (from 74.7% to 84.9%) and diabetes (15.2%–27.5%), and vascular atherosclerotic plaques (arterial and aortic soft/hard plaques from 52.9% to 62.2% and 87.6%–89.3%, respectively). In another recent study, among 11,795 KDPI>85% kidneys recovered for transplant in the U.S., 56.4% of kidneys (*n* = 6,214) were discarded, with biopsy findings (mainly glomerulosclerosis [GS], interstitial fibrosis [IF], arteriosclerosis [AS]) (*n* = 2,747, 44.2%) and unspecified anatomical abnormalities (*n* = 342, 5.5%) reported as reasons for discard [9, 12]. It is expected that transplant decision-makers regularly face assessments of macroscopic (atherosclerotic soft and hard plaques) and microscopic vasculopathy (AS, GS, IF) in older donors with multiple comorbidities. Aortic and renal arterial plaques may make arterial anastomosis challenging and increase the risk for vascular complications (bleeding, thrombosis, and dissection), limit blood flow by causing luminal stenosis, and can adversely affect long-term renal outcomes [5, 13]. In addition, extrinsic atherosclerosis (as manifested by aortic and/or renal artery plaque formation) can result in progression of chronic kidney disease, and could also represent involvement of renal microvasculature [14].

Aging kidneys, typified in ECDs, undergo anatomical and physiological changes as a part of true renal physiological senescence and common diseases (hypertension, diabetes, obesity, atherosclerosis). These changes increase progressively with age and include nephrosclerosis (comprising AS, GS, IF, tubular atrophy and arterial hyalinosis), decline in number of functional glomeruli and glomerular filtration rate (GFR) [15, 16]. Kidney length and total volume remain stable until very old age (>70 years-old), but renal cortical parenchymal volume could be predisposed to decrease with aging [17], hypertension and atherosclerosis [18], and have important implications for inferior renal transplant outcomes, especially, in the setting of small donor kidney length and volume compared to recipient size [4, 19, 20]. Smaller kidney size (length< 10 cm) is associated with older age (decreased nephron mass due nephrosclerosis), shorter height, lower BMI, hypertension and atherosclerosis, while larger kidney size (>12 cm) is generally observed in younger donor age, taller height, higher body mass index (BMI> 30 kg/m^2^), diabetes (hyperfiltration), and congested kidneys (resulting from tissue injury/edema and poor perfusion during deceased donor recovery) [2].

A recent analysis (an abstract presented at the American Transplant Congress in 2022) studied the relationship between kidney anatomy findings (length, severe arterial plaque, hard plaque, cyst/discoloration, infarcted areas, fat cleaned, and subcapsular hematoma) and kidney utilization in a cohort of adult deceased kidney donors with at least one kidney recovered and relevant DonorNet attachments identified using the Organ Procurement and Transplantation Network (OPTN) database in 2019 (*N* = 9,433) [21]. In a multivariable logistic regression adjusted for KDPI and biopsy status, they reported an increased odds of discard with presence of severe arterial plaque (odds ratio [OR] 1.63; 95% confidence interval [CI]: 1.03, 2.59) and hard arterial plaque (OR 2.03; 95% CI: 1.48, 2.80). The authors also showed a U-shaped relationship with kidney length and discard (kidney length [OR 0.36; CI: 0.23, 0.56] and kidney length squared [OR 1.04; CI: 1.02, 1.06]).

In this study, we analyzed the relationship between kidney length and vascular plaque (aortic plaque and arterial plaque) reported in attachments uploaded to DonorNet and 10-year hazard of all-cause graft failure. We hypothesized a nonlinear relationship between kidney length and graft failure risk. We also surmised that the presence and type (hard, soft; aortic, arterial) of vascular plaque would be associated with higher graft failure risk.

## Materials and Methods

We primarily utilized the same study cohort from our previous BARETO study and the materials and method section mirrored those described in the publication [11]. This study used data from the Organ Procurement and Transplantation Network. The OPTN data system includes data on all donors, wait-listed candidates, and transplant recipients in the US, submitted by members of the OPTN. The Health Resources and Services Administration (HRSA), U.S. Department of Health and Human Services, provides oversight to the activities of the OPTN contractor. Data, including DonorNet® attachments, were released to the United Network for Organ Sharing (UNOS) by the OPTN after Institutional Review Board (IRB) approval from Virginia Commonwealth University Ethics Board. The study was therefore been performed in accordance with the ethical standards laid down in an appropriate version of the 2000 Declaration of Helsinki^1^ as well as the Declaration of Istanbul 2008^2^. The IRB granted a waiver of consent due to retrospective observational nature of the analysis.

In the United States, when a patient is diagnosed with brain death in a hospital, donor hospitals collaborate with Organ Procurement Organizations (OPOs) in their respective donor service areas (DSAs). There are over 1,000 donor hospitals and 57 OPOs regulated by the Centers for Medicare and Medicaid Services (CMS). OPOs are responsible for tasks such as obtaining consent, transmitting donor data to the United Network for Organ Sharing (UNOS) through a web portal called UNet, procuring organs, and delivering them to transplant centers. Evaluation of deceased donor kidneys is conducted by surgical recovery teams consisting of transplant surgeons and OPO donation coordinators. OPOs use a platform called DonorNet to upload and modify deceased donor information, including anatomy and biopsy data saved as PDFs. However, there are over 25 different forms used by OPOs for kidney anatomy and pathology reporting, leading to potential variability and subjectivity in the assessment process due to different protocols, expertise levels, and available resources among OPOs. Efforts to standardize the process continue.

DonorNet PDF attachments were manually reviewed and biopsy and anatomy findings entered into the Research Electronic Data Capture (REDCap) [22] database according to a protocol (Supplementary Figure S1) aligned with the Banff Histopathological Consensus criteria [23] for 4,480 extended criteria donors (ECD) recovered from 2008–2012 with at least one kidney reported as having been biopsied and transplanted. Of these, 3,957 (88.3%) had at least one kidney transplanted, and an anatomy attachment found. Among these transplanted donors, 3,006 (76.0%) had both kidneys transplanted, while 951 (24.0%) had just one kidney transplanted. Since the exposure variables in the broader BARETO study include not only anatomy but also biopsy findings, ECD donors, which we found to be almost universally biopsied (93.2%), were chosen to avoid confounding by indication [24] resulting from for cause biopsies [23].

The three anatomy dimensions reported with high frequency on attachments were aortic plaque (99.7% reported), arterial plaque (99.3% reported), and kidney length (99.5% reported). For kidneys with multiple anatomy attachments (1.0% reported), we chose for analysis the attachment with the fewest missing or unknown data elements among these three variables (aortic plaque, arterial plaque, and kidney length). Due to the high correlation between aortic and arterial plaque (Supplementary Tables S1, S2), it was judged infeasible to reliably estimate the independent effect of each type of plaque adjusting for the other. Instead, we created a new exposure variable—the composite plaque score (range 0–4)—by adding the degree of aortic (hard = 2, soft = 1, none = 0) and arterial (hard = 2, soft = 1, none = 0) plaques. The presence of any plaque (yes, no) was also analyzed.

The primary study outcome was all-cause graft failure up to 10 years post-transplant, which was analyzed by the Kaplan-Meier method for aortic plaque, arterial plaque, plaque score, plaque presence, and kidney length. Plaque score, plaque presence, and kidney length were further analyzed using Cox regression and causal inference methods to serve the study’s central aim of characterizing the independent associations between these three exposure variables and long-term graft survival. Our primary findings were derived using doubly robust regression (DRR) [25], which combines the strengths of propensity-score based inverse probability weighting (IPW) [26] and multiple regression to adjust for potential confounding. DRR weights were based on covariate balancing propensity scores (CBPS) [27]. Unadjusted results, as well as results based on IPW alone and multiple regression alone, are provided for comparison. Following Stensrud and Hernan [28], we interpret the hazard ratio estimates from this study as reflecting the weighted average of the true hazard ratios during the 10 years after transplant.

Statistical inference was derived by bootstrapping the entire DRR process, including single-imputation of missing data using the MICE algorithm [29, 30] (Supplementary Figure S2), with 1,000 bootstrap iterations and percentile-based 95% confidence intervals. Supplementary Tables S1–S4 show the degree of missingness for each covariate. Kidney length was modeled both categorically as well as numerically, using a restricted cubic spline to capture nonlinearity. Pointwise (kidney length of 9 cm, 9.5 cm, 10 cm, …, 14 cm) confidence intervals were generated using the bootstrap process.

Potentially confounding covariates were chosen for inclusion by relying on previous literature; subject matter expertise; clinical hypothesis generation; exposure variable vs. covariate correlation analysis; and a philosophy of erring on the side of inclusion while leveraging opportunities for parsimony. A total of seventeen covariates were ultimately included in each set of models: ten donor characteristics; two recipient factors; donor/recipient sex matching; cold ischemic time; pumped (yes/no); kidney length and percent glomerulosclerosis for plaque score and plaque presence; and kidney-specific aortic and arterial plaque for kidney length (Supplementary Tables S1–S4).

Numerical and graphical correlation analysis was used to assess the relationship between kidney length and donor height, weight, BMI, and KDPI. We used R Software Version 4.1.0, including WeightIt, cobalt, CBPS, mice, survival, rms, and lme4 packages.

## Results

### Effect of Aortic Plaque on Outcomes

**FIGURE 1:**
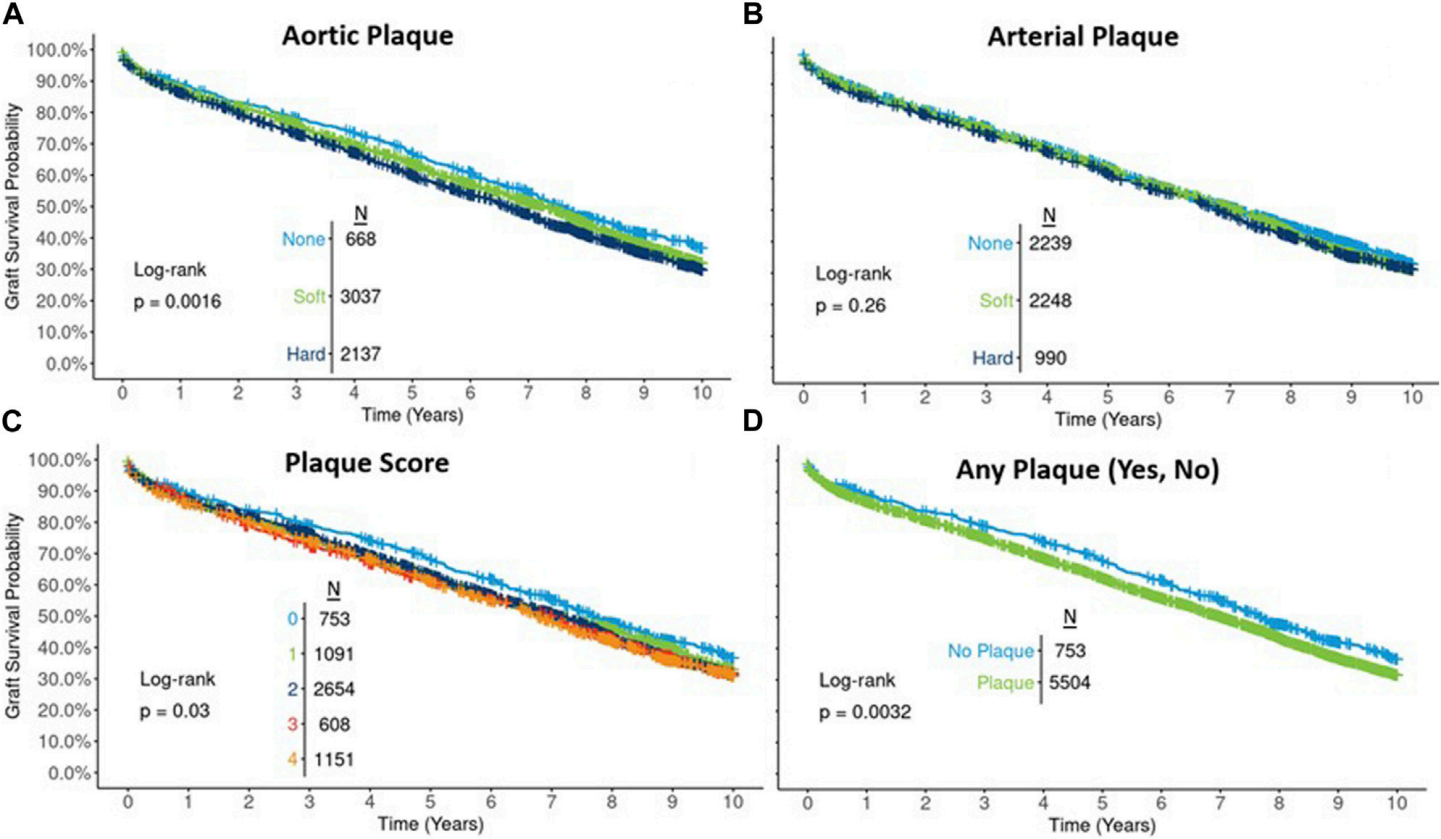
Ten-year Kaplan-Meier graft survival by type of aortic plaque **(A)**, arterial plaque **(B)**, plaque score **(C)**, and presence of any plaque **(D)**.

Unadjusted graft survival based on the Kaplan-Meier method revealed a statistically significant relationship between the degree of aortic plaque (*p* = 0.002, Figure 1A), but not arterial plaque (*p* = 0.26, Figure 1B), and 10-year graft survival. Unadjusted graft survival was significantly lower (*p* = 0.03) for higher plaque score values in an apparent, albeit weak, dose-response relationship (Figure 1C). Similarly, graft survival was significantly lower (*p* = 0.003) for any plaque compared to no plaque (Figure 1D).

Several notable associations were found between plaque score and potentially confounding factors—donor age (*p* < 0.001), KDPI (*p* < 0.001), donor BMI (*p* = 0.03), donor height (*p* < 0.001), donor gender (*p* < 0.001), donor race/ethnicity (*p* < 0.001), donor hypertension (*p* = 0.02), donor diabetes (*p* < 0.001), kidney length (*p* < 0.001), recipient estimated post-transplant survival (EPTS) (*p* < 0.001), cold ischemic time (*p* = 0.001), pumped (*p* < 0.001), donor-recipient mismatch (*p* < 0.001), and percent glomerulosclerosis (*p* < 0.001) (Supplementary Table S3).

**FIGURE 2:**
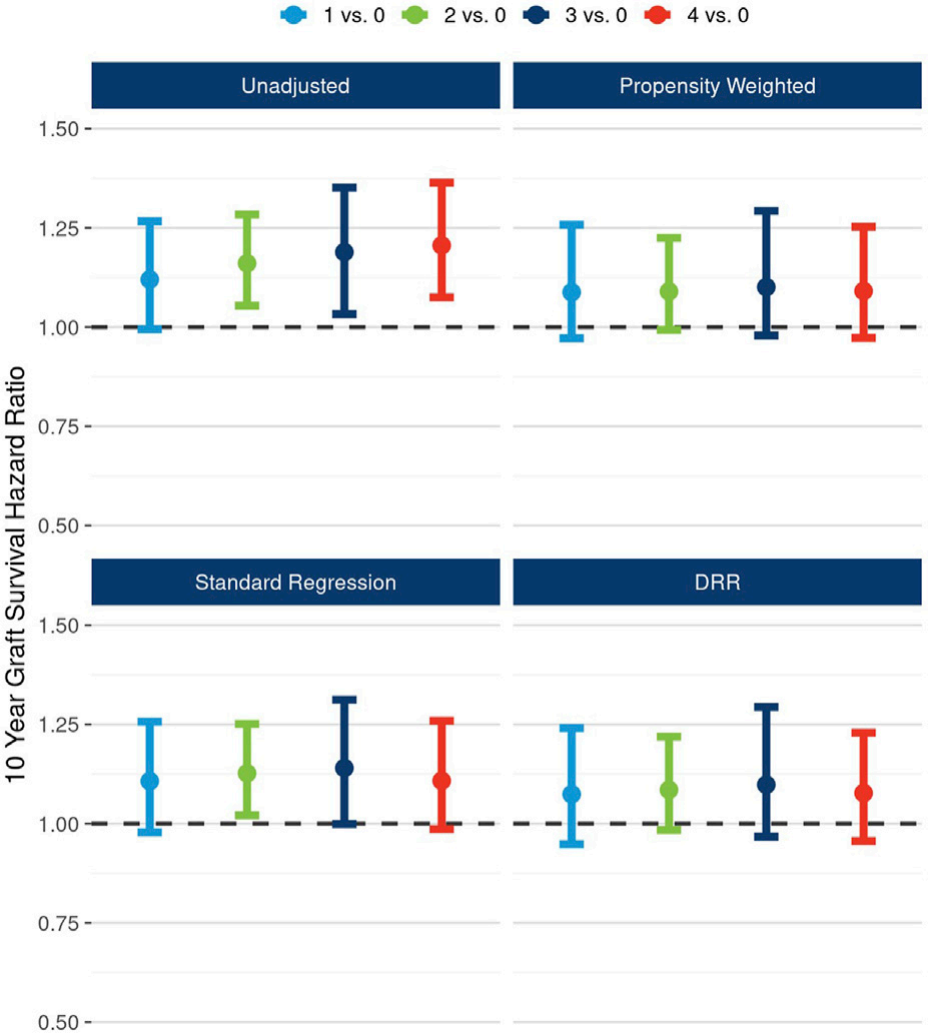
Unadjusted and adjusted (propensity weighted, standard regression, and doubly robust regression -DRR) associations between plaque score and 10-year graft failure risk.

In Cox proportional hazards modeling, after accounting for the associations between plaque score and potential confounders, the 10-year hazard of graft failure for plaque scores 4 vs. 0 attenuated and was no longer significant: unadjusted HR 1.21 (95% CI: 1.08, 1.36), DRR-adjusted HR 1.08 (95% CI: 0.96, 1.23). The mild dose-response relationship evident in the unadjusted results was also attenuated in adjusted analyses (Figure 2).

**FIGURE 3:**
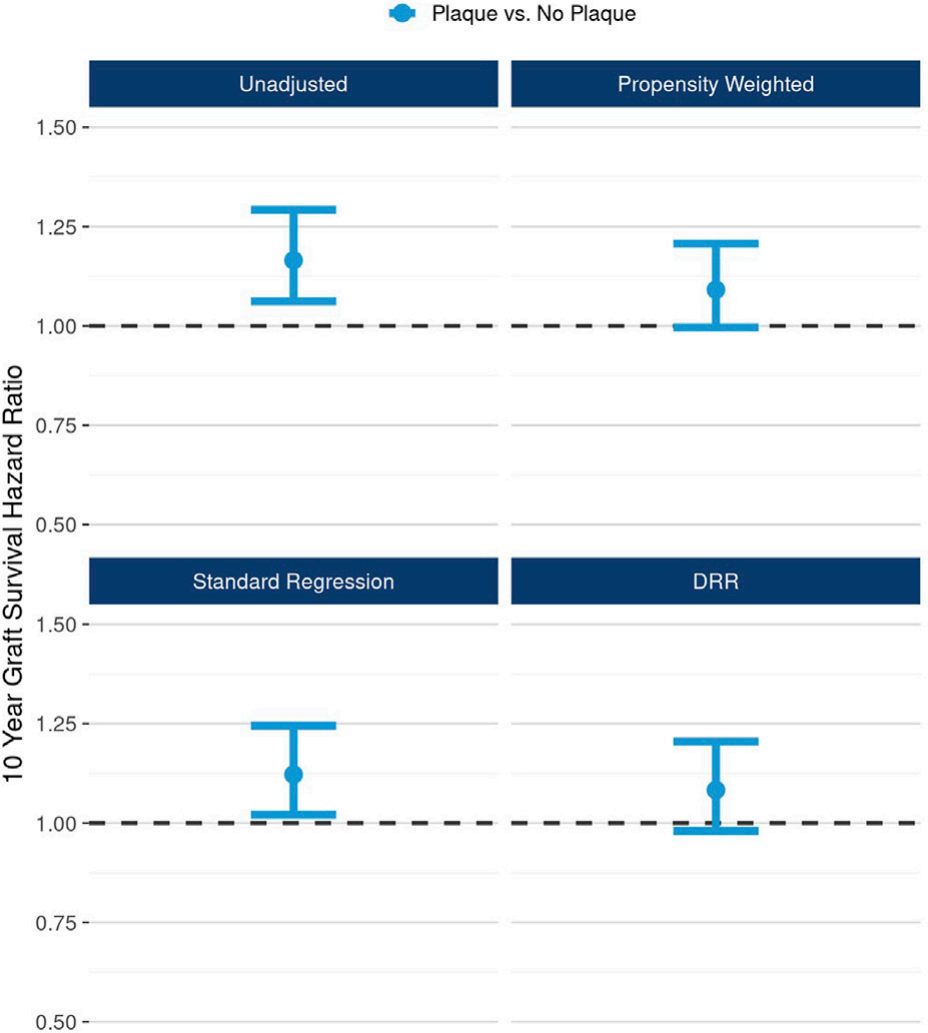
Unadjusted and adjusted (propensity weighted, standard regression, and doubly robust regression -DRR) associations between presence of any plaque and 10-year graft failure risk.

Likewise, after accounting for the associations between the presence of any plaque and potential cofounders, the 10-year hazard of graft failure approached but did not reach statistical significance: unadjusted HR 1.17 (95% CI: 1.06, 1.29), DRR-adjusted HR 1.08 (95% CI: 0.98, 1.20) (Figure 3).

### Effect of Kidney Length on Outcomes

**FIGURE 4:**
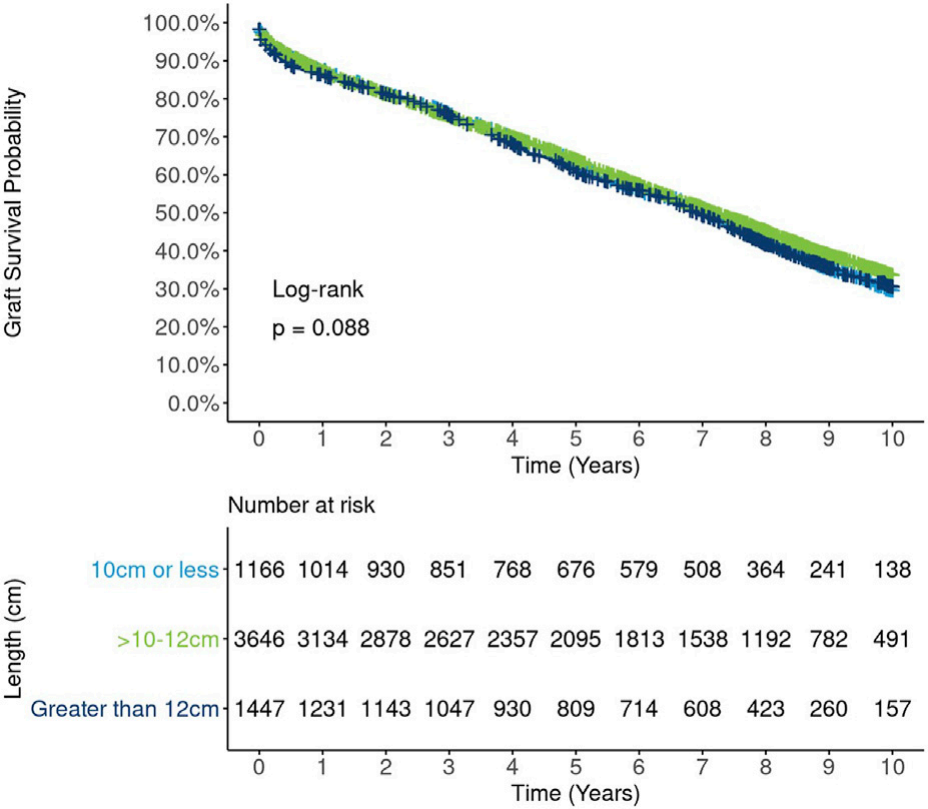
Ten-year Kaplan-Meier graft survival by kidney length.

**FIGURE 5:**
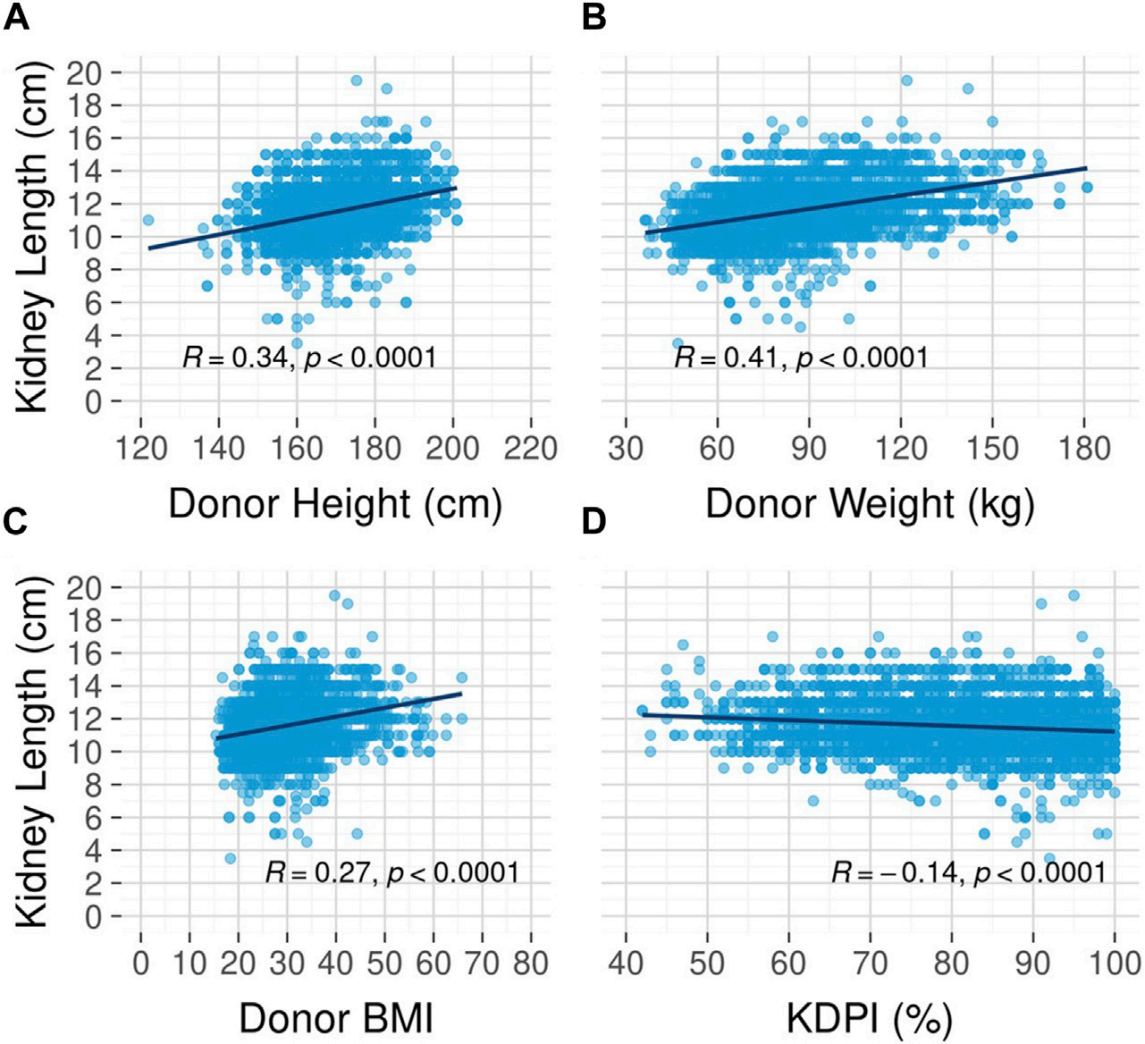
Correlation between kidney length and donor height **(A)**, weight **(B)**, body mass index **(C)**, and kidney donor profile index **(D)**.

Unadjusted survival curves suggest a possible nonlinear relationship between kidney length and long-term graft survival, as the best outcomes were observed for mid-range (10–12 cm) kidneys. However, this relationship did not reach statistical significance (*p* = 0.09, Figure 4). A correlation analysis revealed a moderate to strong positive relationship between kidney length and donor height (Spearman rank correlation coefficient (rho) = 0.34, Figure 5A) and donor weight (rho = 0.41, Figure 5B) and statistically significant but weak correlations between kidney length and donor BMI (rho = 0.27, Figure 5C) and KDPI (rho = −0.14, Figure 5D).

**FIGURE 6:**
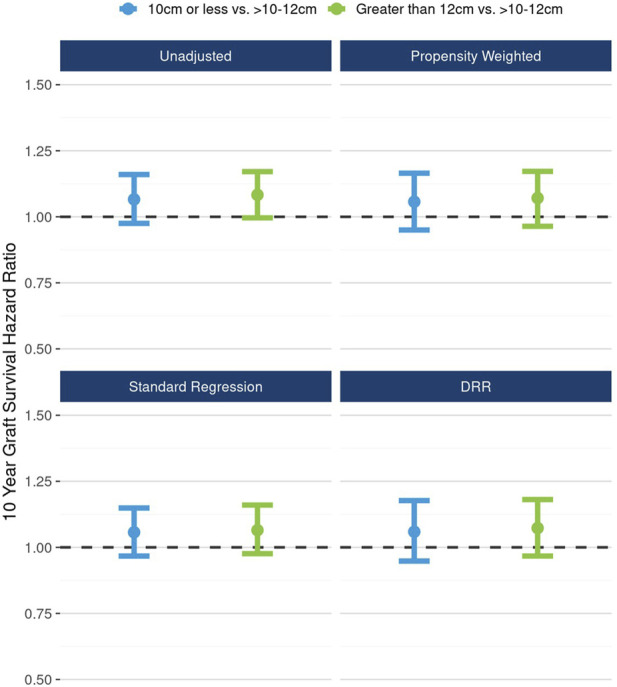
The unadjusted and adjusted (propensity weighted, standard regression, and doubly robust regression -DRR) associations between kidney length and 10-year graft failure risk.

**FIGURE 7:**
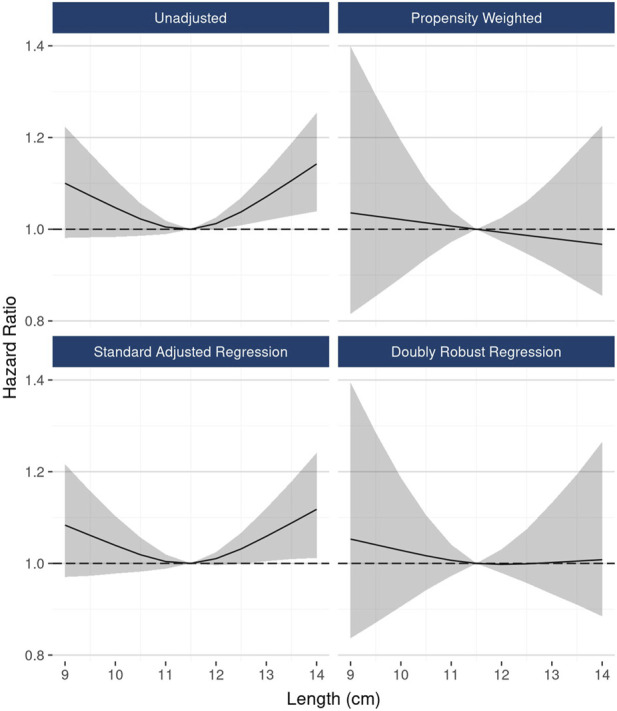
The associations between continuous kidney length and 10-year graft failure risk, modeled as a restricted cubic spline.

In DRR analysis, the hypothesized nonlinear relationship between length and graft survival was still evident, however effects were not statistically significant: ≤10 cm vs.10–12 cm (HR 1.06; 95% CI: 0.98, 1.16), >12 cm vs. 10–12 cm (HR 1.07; 95% CI: 0.97, 1.18) (Figure 6). Unadjusted analysis of continuous kidney length modeled nonlinearly revealed a statistically significant increasing hazard as kidney length rose from about 12 cm to 14 cm; however, this pattern was no longer apparent in fully adjusted DRR analysis, suggesting the nonlinear relationship is, if not entirely, explained by correlations with other donor characteristics (Figure 7).

Love plots [31] (Supplementary Figures S3–S5) indicated highly successful covariate balancing among exposure groups after weighting, with all standardized differences falling near or below 0.1 [32].

## Discussion

After rigorous statistical adjustment for confounding, this study has revealed that the associations between 10-year hazard of graft failure and vascular plaque (analyzed through both a composite plaque score and simply presence vs. absence) approached, but did not reach, statistical significance. The residual effect of plaque presence was significant, suggesting that there may very well be a real, albeit modest, effect that our sample sizes just were not large enough to detect. Still, the key message from this study is that any effect of plaque is small, and thus this parameter should not play a key role in organ utilization decisions. Likewise, the nonlinear relationship that was hypothesized for kidney length and the 10-year hazard of all-cause graft failure indeed manifested, however it is largely or entirely explained by other donor factors, and the residual effects are modest in size and did not reach statistical significance.

The specificity in reporting vascular plaques on the UNOS DonorNet is inadequate. While the descriptions of these plaques are offered in two distinct locations - the aorta and the arteries - and in two types - soft and hard, there is an absence of information regarding their size and extent. This omission hinders differentiation of vascular plaques found in the aortic patch from those located in the renal artery orifice/lumen. In addition, there are currently no objective measurements or standardized scoring system regarding assessment of vascular plaques nor a body of evidence concerning their effects on deceased donor kidney quality and transplant outcomes. Naturally, it is expected that presence of vascular plaque may lead to difficult anastomoses resulting in intra/post-operative complications, such as bleeding and thrombosis, and these plaques can also be interpreted as proxies for intra-renal histological arteriosclerosis.

In one of the few studies published on the topic, Keijback et al. analyzed the data of the kidneys (donor age >50 years old) recovered for transplant (N = 2,610; 2,239 transplanted [85.8%] and 371 discarded [14.2%]) between 2000 and 2015 in the Netherlands as a part of the Eurotransplant system, where renal artery macroscopic arteriosclerosis data were available [5]. Their study revealed that the macroscopic arteriosclerosis commonly occurred, 68% in the transplanted kidneys (none 31%, mild 9%, moderate 46%, massive 13%) and 79% in the discarded kidneys (none 22%, mild 13%, moderate 31%, massive 35%), and increased the risk of discard by 36% (odds ratio [OR], 1.36; 95% confidence interval [CI] 1.02–1.80, *p*-value = 0.03). However, compared to the no vascular lesion category, the macroscopic arteriosclerosis (any degrees) was not associated with delayed graft function (DGF), estimated glomerular filtration rate at 1-year post-transplant, and death censored graft failure during the study period. Early vascular complications leading to graft failure (primary non-function-PNF) among the kidneys with moderate to massive arteriosclerosis were rare and did not differ compared to the kidneys without vascular lesions. These insignificant findings on the outcomes could at least partly be due to small sample size, i.e., insufficient power to detect subtle differences. Among the subgroup of kidney transplant recipients who had a pre-implantation allograft biopsy (n = 109), Keijback et al. showed that there was no correlation between macroscopic renal arteriosclerosis and histological arteriosclerosis (specifically, vascular fibrous intimal thickening and arteriolar hyalinosis). Still, a bias regarding the effect of arteriosclerosis could be introduced in their conclusions because they did not analyze the relationship between macroscopic and microscopic arteriosclerosis correlation among discarded kidneys. In our study, we also observed that the procurement biopsy findings (interstitial fibrosis, vascular changes, and to some extent glomerulosclerosis) did not deteriorate with the presence of higher degree of aortic and arterial plaques (Supplementary Table S1). In turn, we cautiously suggest that vascular plaques should not be viewed as surrogate for intra-renal chronic vascular changes and related histological findings.

The presence of vascular hard stenotic plaques may imply a contraindication to deceased donor transplantation. Depending on the availability of the Carrel aortic patch, the location of hard stenotic plaque (involving ostium and extending into renal artery), length of plaque free renal artery (safe anastomosis generally requiring main renal artery 1.5 cm or longer), either resection of a segment of artery/Carrel patch containing plaque (permitting end to side anastomosis in a similar fashion performed in living donor kidney transplantation) or eversion endarterectomy can be successfully performed as a rescue procedure but requires increased technical expertise [33].

Studies analyzing the relationship between kidney length and short/long-term graft outcomes are limited. Tierie et al. conducted a prospective pilot study (N = 166) to predict the effect of systematic procurement surgical assessment (16 donor variables related to kidney temperature, anatomy [length and width], atherosclerosis, perfusion, and overall quality) on short term graft outcomes (DGF or PNF vs. immediate function, 1-year graft failure or eGFR< 50 mL/min/1.73 m^2^ vs. functioning graft or eGFR>50 mL/min/1.73 m^2^) [2]. In multivariable logistic regression analysis, a larger kidney width (>6 cm) and the poor quality of perfusion (suggestive of congested and edematous kidneys) were associated with DGF/PNF. A larger kidney length (>12 cm), lower first donor creatinine and KDPI predicted a functioning graft or eGFR>50 mL/min/1.73 m^2^ at 1-year. In contrast, our analysis revealed that the kidney length>12 cm was not associated with better long-term graft survival.

Some may have legitimate concern that these donor anatomy parameters are subject to significant measurement errors (kidney length measurement with perinephric fat and lack of kidney volume assessment accounting for the three-dimensional nature of the kidney) and can have subjectivity (lack of length, location, and extension of stenotic hard vascular plaque). However, despite their imperfections, analyzing these parameters’ associations with outcomes is meaningful and relevant since they are used in clinical practice to influence kidney utilization decision making. Moreover, OPTN policy requires transplant centers to update their “Kidney Minimum Acceptance” criteria annually in which the kidney anatomy section includes questions regarding both vascular plaque (considering a kidney from a donor with soft or hard plaque in the renal artery described as mild, moderate, severe) and kidney length (considering a donor kidney that is 2 or more centimeters smaller that the kidney on the opposite site) [34]. Despite known limitations, semi-quantitative assessment is a commonly applied, accepted and undisputable part of the kidney allocation system, as encountered with procurement kidney biopsy reporting, the Banff Histopathological Consensus Criteria for preimplantation kidney biopsies similarly classify IF, tubular atrophy, AS, arteriolar hyalinosis, acute tubular necrosis findings in four categories (none, mild, moderate, and severe).

Evaluating kidney size is a multifaceted process, and it would be erroneous to base it entirely on the measurement of bipolar length for an accurate estimation of kidney volume. One must bear in mind that a kidney of lesser length might compensate with a greater width, hence maintaining a similar overall volume and nearly equivalent split function. Therefore, when sizing a kidney, it’s essential to take into account the substantial disparity in sizes between the two kidneys from the same donor, rather than concentrating exclusively on their total length. This consideration could potentially clarify the observed absence of correlation between length and our outcome of interest.

Ensuring compatibility between the donor kidney size and the metabolic demand of the recipient is vital in kidney transplantation. While there are general guidelines in place, individual factors also play a significant role. Elements like body size, age, and overall health status ought to be considered during the evaluation of kidney size. For instance, a small kidney may not suffice for a large, young recipient due to inadequate kidney function. Conversely, the same small kidney might be appropriate for an older recipient with a reduced body size and metabolic requirement. By taking into account these factors, the transplanted kidney’s capacity to meet the recipient’s needs can be maximized, thereby enhancing the likelihood of a successful transplant outcome.

Our study has strengths and limitations. Using national registry data provided large sample sizes for increased statistical power. We also applied rigorous causal inference methods adjusting for numerous potential confounders. Utilizing DonorNet attachments reflects the real-world framework. Our use of a 2008–2012 cohort allowed us to analyze the effects of kidney length and macroscopic/microscopic vascular disease on 10-year hazard of all-cause graft failure, a meaningful outcome to patients. Even so, it is plausible that unmeasured variables and selection bias related to kidney utilization (transplant vs. discard) may affect the results. Smaller sample sizes for the most extreme values of the three renal anatomy dimensions could have decreased statistical power. The reported data on luminal narrowing in renal artery was not specific (arterial plaque <50%, >50% or circumferential not quantified). Aortic plaque usually involves the distal aorta but sometimes can involve aorta at origin of renal arteries. Presence of aortic plaque at renal artery orifice and its’ extension into renal hilum are also not available. Plaque assessment is a subjective and can vary between surgeons based on experience which may introduce a selection bias. Lastly, we analyzed the effect of individual kidney length measurement on the outcome but not the effect of significant length asymmetry between two mate kidneys.

Despite these limitations, our data suggest that any effect of vascular plaques on the 10-year hazard of all-cause graft failure is small, which should justify a diminished influence on decision-making regarding organ utilization. Secondly, vascular plaques should not be viewed as surrogate for intra-renal chronic vascular histological findings. Finally, though a nonlinear relationship between kidney length and long-term outcomes is evident, it is explained by other pre-measured and reported donor factors and thus should not be ‘double-counted’ when weighing factors in organ acceptance decisions.

Carefully quantifying the independent effects of prognostic parameters on outcomes meaningful to patients and their providers has the potential to improve transplant decision-making and organ utilization. Standardized OPTN data collection on renal anatomy data may improve decision-making and allow for more robust future analyses, like what the OPTN has in the works for biopsy findings like standardized forms and electronic data capture.

## Data Availability

The data analyzed in this study is subject to the following licenses/restrictions: The data are not publicly available due to privacy or ethical restrictions. Requests to access these datasets should be directed to GG, gaurav.gupta@vcuhealth.org.

## Footnotes

1
https://www.wma.net/policies-post/wma-declaration-of-helsinki-ethical-principles-for-medical-research-involving-human-subjects/

2
http://multivu.prnewswire.com/mnr/transplantationsociety/33914/docs/33914-Declaration_of_Istanbul-Lancet.pdf

## Abbreviations

ACGF, all-cause graft failure; AS, arteriosclerosis; BARETO, Biopsy Anatomy & Resistance Effects on Transplant Outcomes; DGF, delayed graft function; DRR, doubly robust regression; ECD, expanded criteria donors; GS, glomerulosclerosis; IF, interstitial fibrosis; IPW, inverse probability weighting; KDPI, Kidney Donor Profile Index; OPTN, Organ Procurement and Transplantation Network; PNF, primary non-function; Redcap, Research Electronic Data Capture; Rho, Spearman rank correlation coefficient; UNOS, United Network for Organ Sharing.
