# Supplementary material for: The Independent Effects of Kidney Length and Vascular Plaque on Ten-Year Outcomes of Extended Criteria Donor Kidney Transplants
Source: Transpl Int. 2023 Jul 14;36:11373. doi: 10.3389/ti.2023.11373 (PMC10379651; doi:10.3389/ti.2023.11373)
Supplement: Supplementary file 1 [file DataSheet1.pdf]

Supplementary Documents:

Supplementary Figures

1. RedCap Biopsy and Anatomy Data Collection Instrument
2. Bootstrapped Doubly Robust Regression Process Flow
3. Love Plot for Plaque Score
4. Love Plot for Plaque Presence
5. Love Plot for Length

Supplementary Tables

1. Associations between Aortic Plaque and Donor/Recipient Characteristics, 2008-2012 Biopsied ECD Kidney Transplants
2. Associations between Arterial Plaque and Donor/Recipient Characteristics, 2008-2012 Biopsied ECD Kidney Transplants
3. Associations between Plaque Score and Modeled Covariates, 2008-2012 Biopsied ECD Kidney Transplants
4. Associations between Kidney Length and Modeled Covariates, 2008-2012 Biopsied ECD Kidney Transplants

Supplementary Figures:

Figure S-1: RedCap Biopsy and Anatomy Data Collection Instrument (see Supplemental Page 25-34 for full version)

Figure S-2. Bootstrapped Doubly Robust Regression Process Flow

Generate bootstrap sample

1. Conditional imputation of missings using chained equations (MICE)
2. Inverse probability of treatment weighting (IPW)
3. Examine covariate balance after weighting (for a sampling of iterations)
4. Run Cox regression 4 ways and store output (hazard ratios (HRs))
   a) Naïve (unadjusted) Cox model
   b) Propensity-score weighted Cox model (no covariates)
   c) Multivariable regression Cox model (covariates)
   d) Doubly robust regression

Repeat 1000 times

Estimate HR point-estimates and percentile-based confidence intervals

Figure S-3. Love Plot for Plaque Score

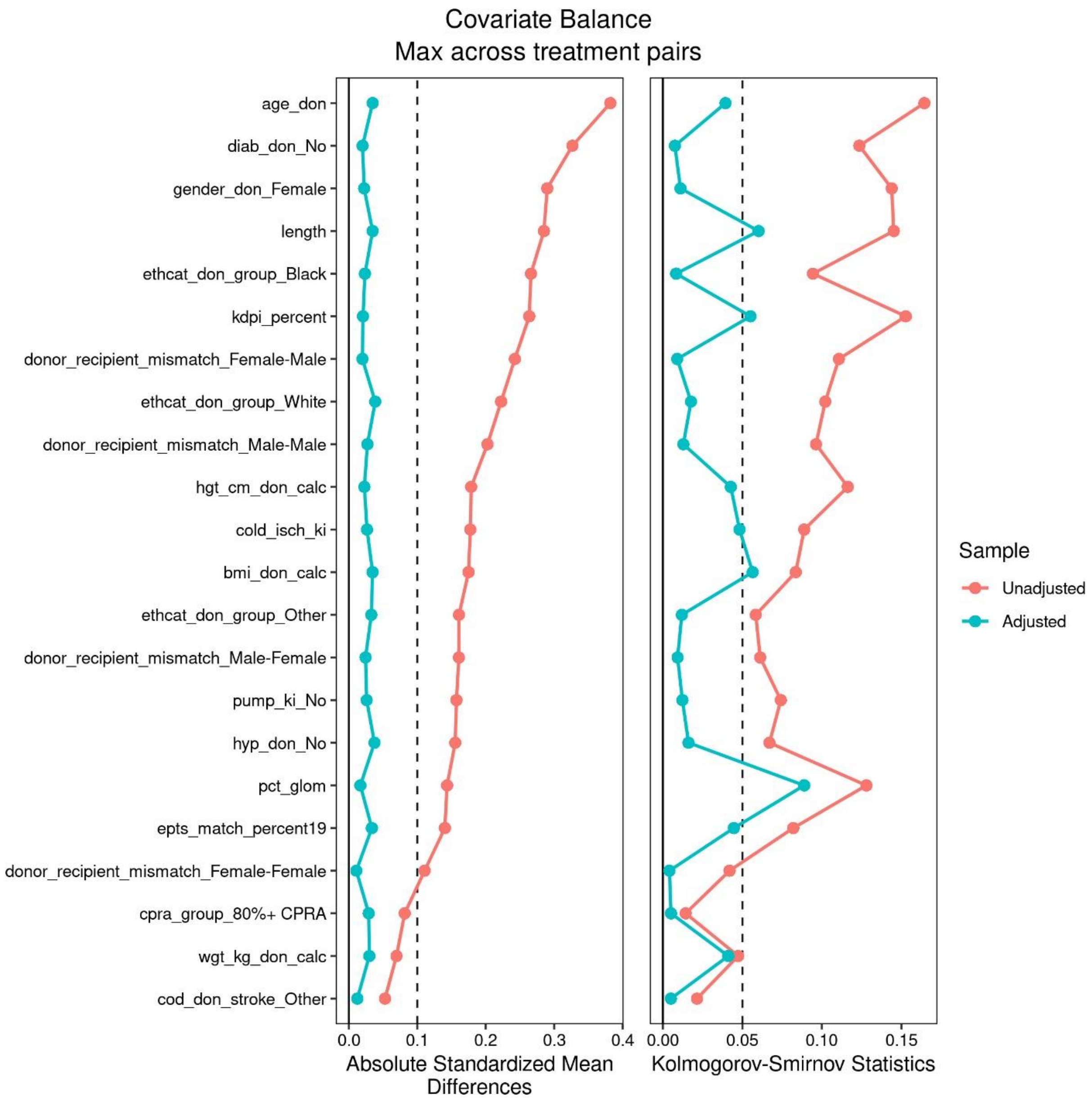

Figure S-4. Love Plot for Plaque Presence (Yes, No)

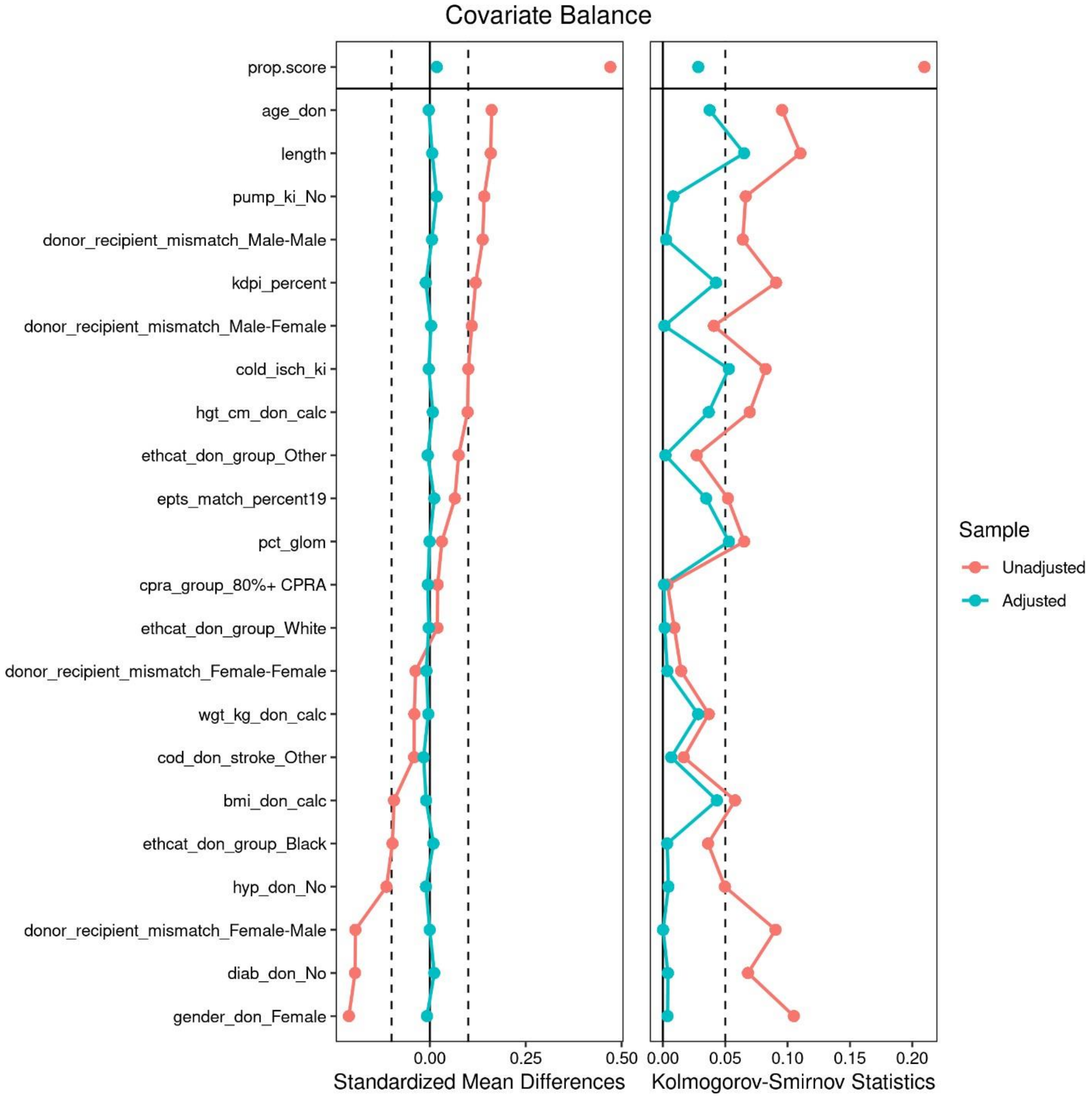

Figure S-5. Love Plot for Kidney Length

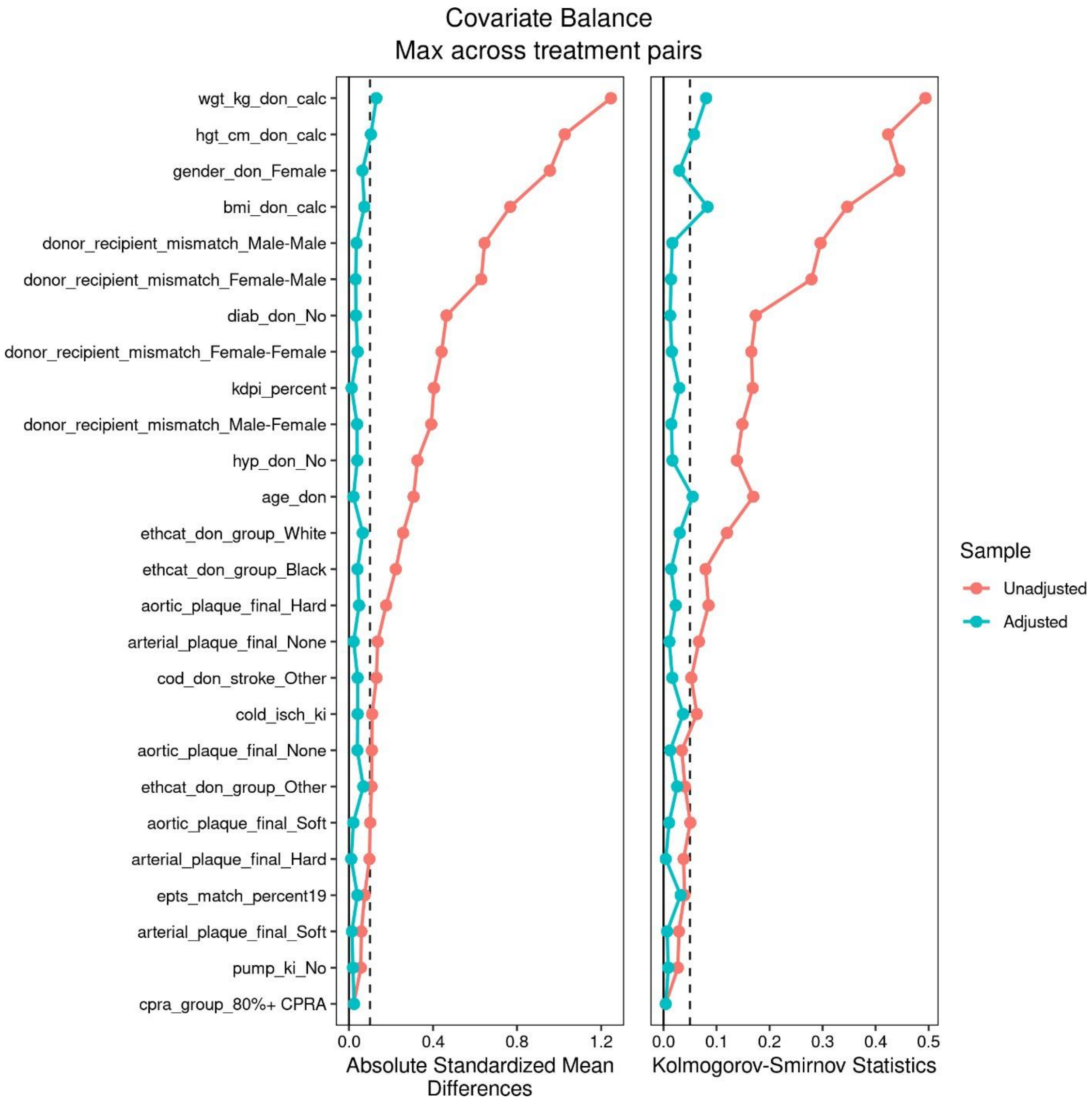

Supplementary Tables:

Supplementary Table S-1. Associations between Aortic Plaque and Donor/Recipient Characteristics, 2008-2012 Biopsied ECD Kidney Transplants

	Aortic Plaque			
	None (N=668)	Soft (N=3037)	Hard (N=2137)	p value
**Donor Age**				<0.0001[1]
N	668	3037	2137	
Missing	0	0	0	
Mean (SD)	58.9 (5.6)	59.3 (6.0)	60.7 (6.0)	
**KDPI**				<0.0001[1]
N	656	3016	2117	
Missing	12	21	20	
Mean (SD)	80.4 (11.1)	81.3 (11.4)	82.8 (11.1)	
**Donor BMI**				0.0006[1]
N	666	3035	2132	
Missing	2	2	5	
Mean (SD)	29.7 (6.9)	29.3 (6.6)	28.6 (6.0)	
**Donor Height (cm)**				<0.0001[1]
N	668	3037	2137	
Missing	0	0	0	
Mean (SD)	169.5 (9.8)	170.0 (10.3)	171.1 (9.9)	
**Donor Weight (kg)**				0.4171[1]

	None (N=668)	Soft (N=3037)	Hard (N=2137)	p value
N	667	3035	2137	
Missing	1	2	0	
Mean (SD)	85.3 (20.9)	84.7 (20.8)	83.7 (19.4)	
**Donor Gender**				<0.0001[2]
Female	372 (55.7%)	1453 (47.8%)	924 (43.2%)	
Male	296 (44.3%)	1584 (52.2%)	1213 (56.8%)	
**Donor Cause of Death**				0.0232[2]
Cerebrovascular/Stroke	513 (76.8%)	2447 (80.6%)	1668 (78.1%)	
Other	155 (23.2%)	590 (19.4%)	469 (21.9%)	
**Donor Race/Ethnicity**				<0.0001[2]
White	442 (66.2%)	1918 (63.2%)	1588 (74.3%)	
Black	137 (20.5%)	529 (17.4%)	238 (11.1%)	
Other	89 (13.3%)	590 (19.4%)	311 (14.6%)	
**Donor History of Hypertension**				0.0031[2]
Missing	7 (.%)	12 (.%)	8 (.%)	
Yes	474 (71.7%)	2255 (74.5%)	1652 (77.6%)	
No	187 (28.3%)	770 (25.5%)	477 (22.4%)	
**Donor Diabetes**				<0.0001[2]
Missing	5 (.%)	18 (.%)	17 (.%)	
Yes	74 (11.2%)	498 (16.5%)	454 (21.4%)	
No	589 (88.8%)	2521 (83.5%)	1666 (78.6%)	

	None (N=668)	Soft (N=3037)	Hard (N=2137)	p value
**Kidney Length (cm)**				<0.0001[1]
N	654	3033	2131	
Missing	14	4	6	
Mean (SD)	11.4 (1.5)	11.5 (1.3)	11.7 (1.4)	
**Arterial Plaque**				<0.0001[2]
Missing	15 (.%)	284 (.%)	227 (.%)	
Hard	7 (1.1%)	54 (2.0%)	918 (48.1%)	
Soft	29 (4.4%)	1637 (59.5%)	554 (29.0%)	
None	617 (94.5%)	1062 (38.6%)	438 (22.9%)	
**EPTS**				0.0420[1]
N	659	3010	2121	
Missing	9	27	16	
Mean (SD)	63.7 (26.4)	65.4 (25.3)	66.7 (25.1)	
**CPRA Group**				0.6090[2]
<80% CPRA	646 (96.7%)	2951 (97.2%)	2067 (96.7%)	
80%+ CPRA	22 (3.3%)	86 (2.8%)	70 (3.3%)	
**Cold Ischemic Time**				<0.0001[1]
N	649	2986	2099	
Missing	19	51	38	
Mean (SD)	18.0 (9.0)	18.6 (8.7)	19.7 (9.2)	

	None (N=668)	Soft (N=3037)	Hard (N=2137)	p value
**Pumped by OPO or TXC (OPTN Data)**				0.0010[2]
Yes	470 (70.4%)	1930 (63.5%)	1428 (66.8%)	
No	198 (29.6%)	1107 (36.5%)	709 (33.2%)	
**Donor-Recipient Mismatch**				<0.0001[2]
Female-Female	123 (18.4%)	523 (17.2%)	368 (17.2%)	
Female-Male	249 (37.3%)	930 (30.6%)	556 (26.0%)	
Male-Female	93 (13.9%)	552 (18.2%)	404 (18.9%)	
Male-Male	203 (30.4%)	1032 (34.0%)	809 (37.9%)	
**Percent Glomerulosclerosis**				0.0014[1]
N	615	2808	1949	
Missing	53	229	188	
Mean (SD)	5.6 (7.4)	5.9 (6.8)	6.3 (6.8)	
**Interstitial Fibrosis**				0.5097[2]
Missing	93 (.%)	432 (.%)	345 (.%)	
Absent/Minimal (0-5%)	355 (61.7%)	1540 (59.1%)	1072 (59.8%)	
Mild (6-25%)	202 (35.1%)	997 (38.3%)	663 (37.0%)	
Mild-moderate (26-50%)/Severe (>50%)	18 (3.1%)	68 (2.6%)	57 (3.2%)	
**Chronic Vascular Changes**				0.2800[2]
Missing	135 (.%)	690 (.%)	530 (.%)	
Absent/Minimal (0%)	287 (53.8%)	1145 (48.8%)	807 (50.2%)	
Mild (1-25%)	193 (36.2%)	963 (41.0%)	645 (40.1%)	

	None (N=668)	Soft (N=3037)	Hard (N=2137)	p value
Mild-moderate (26-50%)/Severe (>50%)	53 (9.9%)	239 (10.2%)	155 (9.6%)	
Mean (SD)	5.8 (1.1)	5.7 (1.0)	5.8 (1.0)	

(report generated on 29APR2021)

[1]Kruskal Wallis [2]Chi-Square

Supplementary Table S-2. Associations between Arterial Plaque and Donor/Recipient Characteristics, 2008-2012 Biopsied ECD Kidney Transplants

	Arterial Plaque			
	None (N=2239)	Soft (N=2248)	Hard (N=990)	p value
**Donor Age**				<0.0001[1]
N	2239	2248	990	
Missing	0	0	0	
Mean (SD)	59.3 (5.8)	59.5 (6.0)	61.1 (6.0)	
**KDPI**				0.0005[1]
N	2218	2231	977	
Missing	21	17	13	
Mean (SD)	81.3 (11.6)	81.3 (11.1)	82.8 (10.9)	
**Donor BMI**				0.0022[1]
N	2235	2245	988	
Missing	4	3	2	
Mean (SD)	29.2 (6.7)	29.2 (6.3)	28.3 (6.0)	
**Donor Height (cm)**				0.0017[1]
N	2239	2248	990	
Missing	0	0	0	
Mean (SD)	169.7 (10.0)	170.4 (10.2)	171.0 (9.9)	
**Donor Weight (kg)**				0.0805[1]
N	2238	2246	990	

	None (N=2239)	Soft (N=2248)	Hard (N=990)	p value
Missing	1	2	0	
Mean (SD)	84.3 (20.8)	85.1 (20.2)	82.9 (19.4)	
**Donor Gender**				<0.0001[2]
Female	1163 (51.9%)	1034 (46.0%)	433 (43.7%)	
Male	1076 (48.1%)	1214 (54.0%)	557 (56.3%)	
**Donor Cause of Death**				0.0329[2]
Cerebrovascular/Stroke	1752 (78.2%)	1817 (80.8%)	766 (77.4%)	
Other	487 (21.8%)	431 (19.2%)	224 (22.6%)	
**Donor Race/Ethnicity**				<0.0001[2]
White	1520 (67.9%)	1466 (65.2%)	755 (76.3%)	
Black	412 (18.4%)	327 (14.5%)	93 (9.4%)	
Other	307 (13.7%)	455 (20.2%)	142 (14.3%)	
**Donor History of Hypertension**				0.3095[2]
Missing	13 (.%)	9 (.%)	5 (.%)	
Yes	1659 (74.5%)	1686 (75.3%)	759 (77.1%)	
No	567 (25.5%)	553 (24.7%)	226 (22.9%)	
**Donor Diabetes**				0.0051[2]
Missing	11 (.%)	16 (.%)	11 (.%)	
Yes	353 (15.8%)	380 (17.0%)	201 (20.5%)	
No	1875 (84.2%)	1852 (83.0%)	778 (79.5%)	

	None (N=2239)	Soft (N=2248)	Hard (N=990)	p value
**Kidney Length (cm)**				<0.0001[1]
N	2220	2244	987	
Missing	19	4	3	
Mean (SD)	11.4 (1.4)	11.5 (1.3)	11.7 (1.4)	
**Aortic Plaque**				<0.0001[2]
Missing	122 (.%)	28 (.%)	11 (.%)	
Hard	438 (20.7%)	554 (25.0%)	918 (93.8%)	
Soft	1062 (50.2%)	1637 (73.7%)	54 (5.5%)	
None	617 (29.1%)	29 (1.3%)	7 (0.7%)	
**EPTS**				0.0023[1]
N	2214	2226	984	
Missing	25	22	6	
Mean (SD)	64.1 (25.8)	66.2 (25.2)	67.3 (24.8)	
**CPRA Group**				0.3239[2]
<80% CPRA	2166 (96.7%)	2187 (97.3%)	954 (96.4%)	
80%+ CPRA	73 (3.3%)	61 (2.7%)	36 (3.6%)	
**Cold Ischemic Time**				0.0003[1]
N	2192	2208	972	
Missing	47	40	18	
Mean (SD)	18.7 (8.9)	18.6 (8.7)	20.0 (9.4)	
**Pumped by OPO or TXC (OPTN Data)**				<0.0001[2]

	None (N=2239)	Soft (N=2248)	Hard (N=990)	p value
Yes	1547 (69.1%)	1349 (60.0%)	665 (67.2%)	
No	692 (30.9%)	899 (40.0%)	325 (32.8%)	
**Donor-Recipient Mismatch**				0.0001[2]
Female-Female	435 (19.4%)	367 (16.3%)	164 (16.6%)	
Female-Male	728 (32.5%)	667 (29.7%)	269 (27.2%)	
Male-Female	345 (15.4%)	426 (19.0%)	198 (20.0%)	
Male-Male	731 (32.6%)	788 (35.1%)	359 (36.3%)	
**Donor Terminal Creatinine**				0.1904[1]
N	2239	2248	990	
Missing	0	0	0	
Mean (SD)	1.1 (0.6)	1.2 (0.6)	1.1 (0.6)	
**Percent Glomerulosclerosis**				0.0001[1]
N	2055	2042	912	
Missing	184	206	78	
Mean (SD)	5.6 (6.7)	6.1 (7.0)	6.6 (7.4)	
**Interstitial Fibrosis**				0.1600[2]
Missing	350 (.%)	371 (.%)	149 (.%)	
Absent/Minimal (0-5%)	1181 (62.5%)	1122 (59.8%)	488 (58.0%)	
Mild (6-25%)	652 (34.5%)	700 (37.3%)	322 (38.3%)	
Mild-moderate (26-50%)/Severe (>50%)	56 (3.0%)	55 (2.9%)	31 (3.7%)	

	None (N=2239)	Soft (N=2248)	Hard (N=990)	p value
**Chronic Vascular Changes**				0.6849[2]
Missing	567 (.%)	529 (.%)	223 (.%)	
Absent/Minimal (0%)	875 (52.3%)	900 (52.4%)	399 (52.0%)	
Mild (1-25%)	644 (38.5%)	637 (37.1%)	291 (37.9%)	
Mild-moderate (26-50%)/Severe (>50%)	153 (9.2%)	182 (10.6%)	77 (10.0%)	

(report generated on 29APR2021)
[1]Kruskal Wallis [2]Chi-Square

Supplementary Table S-3. Associations between Plaque Score and Modeled Covariates, 2008-2012 Biopsied ECD Kidney Transplants

	Plaque Score					
	0 (N=753)	1 (N=1091)	2 (N=2654)	3 (N=608)	4 (N=1151)	p value
**Donor Age**						<0.0001[1]
N	753	1091	2654	608	1151	
Missing	0	0	0	0	0	
Mean (SD)	58.7 (5.5)	59.1 (5.8)	59.6 (6.0)	60.3 (6.0)	61.0 (6.0)	
**KDPI**						<0.0001[1]
N	743	1080	2640	601	1138	
Missing	10	11	14	7	13	
Mean (SD)	80.1 (11.2)	81.4 (11.7)	81.6 (11.3)	81.5 (11.1)	83.0 (11.0)	
**Donor BMI**						0.0271[1]
N	751	1091	2650	607	1149	
Missing	2	0	4	1	2	
Mean (SD)	29.5 (6.9)	29.2 (6.5)	29.2 (6.6)	28.6 (5.7)	28.4 (5.9)	
**Donor Height (cm)**						<0.0001[1]
N	753	1091	2654	608	1151	
Missing	0	0	0	0	0	
Mean (SD)	169.5 (9.7)	169.7 (10.3)	170.3 (10.1)	171.3 (10.1)	171.2 (9.8)	
**Donor Weight (kg)**						0.8830[1]
N	752	1091	2652	608	1151	

	Plaque Score					
	0 (N=753)	1 (N=1091)	2 (N=2654)	3 (N=608)	4 (N=1151)	p value
Missing	1	0	2	0	0	
Mean (SD)	84.9 (21.0)	84.3 (21.0)	84.8 (20.6)	84.0 (19.1)	83.5 (19.2)	
**Donor Gender**						<0.0001[2]
Female	425 (56.4%)	542 (49.7%)	1238 (46.6%)	257 (42.3%)	484 (42.1%)	
Male	328 (43.6%)	549 (50.3%)	1416 (53.4%)	351 (57.7%)	667 (57.9%)	
**Donor Cause of Death**						0.6043[2]
Cerebrovascular/Stroke	593 (78.8%)	859 (78.7%)	2123 (80.0%)	486 (79.9%)	896 (77.8%)	
Other	160 (21.2%)	232 (21.3%)	531 (20.0%)	122 (20.1%)	255 (22.2%)	
**Donor Race/Ethnicity**						<0.0001[2]
White	508 (67.5%)	714 (65.4%)	1712 (64.5%)	453 (74.5%)	860 (74.7%)	
Black	144 (19.1%)	211 (19.3%)	431 (16.2%)	63 (10.4%)	114 (9.9%)	
Other	101 (13.4%)	166 (15.2%)	511 (19.3%)	92 (15.1%)	177 (15.4%)	
**Donor History of Hypertension**						0.0206[2]
Missing	4 (.%)	9 (.%)	7 (.%)	4 (.%)	3 (.%)	
Yes	534 (71.3%)	820 (75.8%)	1984 (75.0%)	459 (76.0%)	896 (78.0%)	
No	215 (28.7%)	262 (24.2%)	663 (25.0%)	145 (24.0%)	252 (22.0%)	
**Donor Diabetes**						<0.0001[2]
Missing	5 (.%)	4 (.%)	17 (.%)	4 (.%)	12 (.%)	
Yes	78 (10.4%)	194 (17.8%)	432 (16.4%)	120 (19.9%)	251 (22.0%)	

	Plaque Score					
	0 (N=753)	1 (N=1091)	2 (N=2654)	3 (N=608)	4 (N=1151)	p value
No	670 (89.6%)	893 (82.2%)	2205 (83.6%)	484 (80.1%)	888 (78.0%)	
**Kidney Length (cm)**						<0.0001[1]
N	737	1089	2649	606	1148	
Missing	16	2	5	2	3	
Mean (SD)	11.3 (1.5)	11.4 (1.4)	11.5 (1.3)	11.6 (1.3)	11.7 (1.4)	
**EPTS**						0.0087[1]
N	742	1080	2633	602	1144	
Missing	11	11	21	6	7	
Mean (SD)	64.0 (26.3)	63.6 (25.9)	66.0 (24.9)	66.3 (25.6)	67.2 (25.0)	
**CPRA Group**						0.4828[2]
<80% CPRA	725 (96.3%)	1059 (97.1%)	2567 (96.7%)	594 (97.7%)	1108 (96.3%)	
80%+ CPRA	28 (3.7%)	32 (2.9%)	87 (3.3%)	14 (2.3%)	43 (3.7%)	
**Cold Ischemic Time**						0.0006[1]
N	734	1066	2611	595	1131	
Missing	19	25	43	13	20	
Mean (SD)	18.4 (9.1)	18.7 (8.7)	18.8 (8.7)	19.3 (9.0)	20.0 (9.4)	
**Pumped by OPO or TXC (OPTN Data)**						0.0001[2]
Yes	524 (69.6%)	744 (68.2%)	1650 (62.2%)	394 (64.8%)	769 (66.8%)	
No	229 (30.4%)	347 (31.8%)	1004 (37.8%)	214 (35.2%)	382 (33.2%)	

	Plaque Score					
	0 (N=753)	1 (N=1091)	2 (N=2654)	3 (N=608)	4 (N=1151)	p value
**Donor-Recipient Mismatch**						<0.0001[2]
Female-Female	148 (19.7%)	195 (17.9%)	472 (17.8%)	94 (15.5%)	188 (16.3%)	
Female-Male	277 (36.8%)	347 (31.8%)	766 (28.9%)	163 (26.8%)	296 (25.7%)	
Male-Female	107 (14.2%)	176 (16.1%)	502 (18.9%)	114 (18.8%)	234 (20.3%)	
Male-Male	221 (29.3%)	373 (34.2%)	914 (34.4%)	237 (39.0%)	433 (37.6%)	
**Percent Glomerulosclerosis**						0.0001[1]
N	689	1014	2435	548	1067	
Missing	64	77	219	60	84	
Mean (SD)	5.4 (7.3)	5.6 (6.4)	6.0 (6.8)	6.0 (6.8)	6.5 (7.2)	

(report generated on 29APR2021)

[1]Kruskal Wallis [2]Chi-Square

Supplementary Table S-4. Associations between Kidney Length and Modeled Covariates, 2008-2012 Biopsied ECD Kidney Transplants

	Kidney Length			
	10cm or less (N=1166)	>10-12cm (N=3646)	Greater than 12cm (N=1447)	p value
**Donor Age**				<0.0001[1]
N	1166	3646	1447	
Missing	0	0	0	
Mean (SD)	60.6 (6.2)	59.8 (6.0)	58.8 (5.6)	
**KDPI**				<0.0001[1]
N	1154	3618	1432	
Missing	12	28	15	
Mean (SD)	84.1 (10.6)	81.6 (11.2)	79.6 (11.8)	
**Donor BMI**				<0.0001[1]
N	1165	3640	1445	
Missing	1	6	2	
Mean (SD)	26.6 (5.7)	28.9 (6.3)	31.4 (6.6)	
**Donor Height (cm)**				<0.0001[1]
N	1166	3646	1447	
Missing	0	0	0	
Mean (SD)	165.4 (8.9)	170.1 (9.7)	175.1 (9.8)	
**Donor Weight (kg)**				<0.0001[1]
N	1166	3645	1445	

	Kidney Length			
	10cm or less (N=1166)	>10-12cm (N=3646)	Greater than 12cm (N=1447)	p value
Missing	0	1	2	
Mean (SD)	72.8 (16.2)	83.5 (18.7)	96.3 (21.2)	
**Donor Gender**				<0.0001[2]
Female	808 (69.3%)	1779 (48.8%)	359 (24.8%)	
Male	358 (30.7%)	1867 (51.2%)	1088 (75.2%)	
**Donor Cause of Death**				0.0010[2]
Cerebrovascular/Stroke	970 (83.2%)	2861 (78.5%)	1128 (78.0%)	
Other	196 (16.8%)	785 (21.5%)	319 (22.0%)	
**Donor Race/Ethnicity**				<0.0001[2]
White	730 (62.6%)	2431 (66.7%)	1079 (74.6%)	
Black	210 (18.0%)	613 (16.8%)	146 (10.1%)	
Other	226 (19.4%)	602 (16.5%)	222 (15.3%)	
**Donor History of Hypertension**				<0.0001[2]
Missing	3 (.%)	17 (.%)	7 (.%)	
Yes	810 (69.6%)	2686 (74.0%)	1202 (83.5%)	
No	353 (30.4%)	943 (26.0%)	238 (16.5%)	
**Donor Diabetes**				<0.0001[2]
Missing	12 (.%)	22 (.%)	8 (.%)	
Yes	117 (10.1%)	566 (15.6%)	396 (27.5%)	

	Kidney Length			
	10cm or less (N=1166)	>10-12cm (N=3646)	Greater than 12cm (N=1447)	p value
No	1037 (89.9%)	3058 (84.4%)	1043 (72.5%)	
**Arterial Plaque**				0.0062[2]
Missing	136 (.%)	470 (.%)	202 (.%)	
Hard	180 (17.5%)	548 (17.3%)	259 (20.8%)	
Soft	398 (38.6%)	1326 (41.8%)	520 (41.8%)	
None	452 (43.9%)	1302 (41.0%)	466 (37.4%)	
**Aortic Plaque**				<0.0001[2]
Missing	90 (.%)	264 (.%)	87 (.%)	
Hard	362 (33.6%)	1194 (35.3%)	575 (42.3%)	
Soft	579 (53.8%)	1794 (53.0%)	660 (48.5%)	
None	135 (12.5%)	394 (11.6%)	125 (9.2%)	
**EPTS**				0.1390[1]
N	1160	3610	1433	
Missing	6	36	14	
Mean (SD)	65.1 (25.3)	65.3 (25.7)	66.9 (24.5)	
**CPRA Group**				0.7670[2]
<80% CPRA	1128 (96.7%)	3523 (96.6%)	1404 (97.0%)	
80%+ CPRA	38 (3.3%)	123 (3.4%)	43 (3.0%)	
**Cold Ischemic Time**				0.0033[1]

	Kidney Length			
	10cm or less (N=1166)	>10-12cm (N=3646)	Greater than 12cm (N=1447)	p value
N	1145	3575	1418	
Missing	21	71	29	
Mean (SD)	18.7 (8.9)	18.9 (9.0)	19.7 (8.9)	
**Pumped by OPO or TXC (OPTN Data)**				0.2198[2]
Yes	736 (63.1%)	2400 (65.8%)	934 (64.5%)	
No	430 (36.9%)	1246 (34.2%)	513 (35.5%)	
**Donor-Recipient Mismatch**				<0.0001[2]
Female-Female	302 (25.9%)	656 (18.0%)	135 (9.3%)	
Female-Male	506 (43.4%)	1123 (30.8%)	224 (15.5%)	
Male-Female	128 (11.0%)	629 (17.3%)	374 (25.8%)	
Male-Male	230 (19.7%)	1238 (34.0%)	714 (49.3%)	

(report generated on 29APR2021)
[1]Kruskal Wallis [2]Chi-Square

VCU Biopsy Study Data Collection

Figure S-1: REDCap Biopsy and Anatomy Data Collection Instrument

Record ID

OPTN Donor Id
* must provide value
Type initial letters to filter Donor ID dropdown

Confirm OPTN Donor Id
* must provide value

Kidney Laterality
* must provide value

Biopsy Information

Was a Biopsy Report found among the attachments?
* must provide value
- ○ Yes
- ○ No

reset

Type of Biopsy Sample:
* must provide value
- ○ Needle / Core
- ○ Wedge
- ○ Other
- ○ Unknown

reset

Other Biopsy Sample Type:
* must provide value

Expand

Biopsy Preparation Method:
* must provide value
- ○ Frozen section
- ○ Fixed/permanent (parain-embedded)
- ○ Other
- ○ Unknown

reset

Other Biopsy Sample Preparation Method:
* must provide value

Expand

Number of Glomeruli Observed:

Number of Glomeruli Sclerosed:

% Glomerulosclerosis (Calculated Field): View equation

% Glomerulosclerosis (Direct Entry of Percentage)

Interstitial Fibrosis*:

(* Note: absent and minimal will be grouped together for analysis, so the distinction is not critical.)

* must provide value

- ◯ Absent/none (0%)
- ◯ Minimal (1-5%)
- ◯ Mild (6-25%)
- ◯ Mild-moderate (26-50%)
- ◯ Severe (>50% of cortex involved)
- ◯ Other
- ◯ Unknown

reset

You entered "Other" for Interstitial Fibrosis. Please describe.

* must provide value

Expand

Arterial intimal fibrosis (aka, arteriosclerosis or chronic vascular changes or vascular damage or vascular narrowing):*

(* Note: absent and minimal will be grouped together for analysis, so the distinction is not important.)

* must provide value

- ◯ Absent/none (0%)
- ◯ Minimal (0%)
- ◯ Mild (1-25%)
- ◯ Mild-moderate (26-50%)
- ◯ Severe (>50% vascular narrowing)
- ◯ Other
- ◯ Unknown

reset

You entered "Other" for Arterial intimal fibrosis (aka, arteriosclerosis or chronic vascular changes or vascular damage or vascular narrowing):. Please describe.

* must provide value

Expand

Arteriolar hyalinosis (strictly involves arterioles, the vessels without internal elastic lamina):*

(* Note: absent and minimal will be grouped together for analysis, so the distinction is not important.)

* must provide value

- ◯ Absent/none
- ◯ Minimal
- ◯ Mild (at least one arteriole)
- ◯ Mild-moderate (more than one arteriole)
- ◯ Severe (multiple arterioles affected, circumferential)
- ◯ Other
- ◯ Unknown

reset

You entered "Other" for Arteriolar hyalinosis (strictly involves arterioles, the vessels without internal elastic lamina). Please describe.

* must provide value

Expand

Tubular atrophy*:

(* Note: absent and minimal will be grouped together for analysis, so the distinction is not critical.)

* must provide value

- ◯ Absent/none (0%)
- ◯ Minimal (0%)
- ◯ Mild (1-25%)
- ◯ Mild-moderate (26-50%)
- ◯ Severe (>50% of cortical tubules involved)
- ◯ Other
- ◯ Unknown

reset

You entered "Other" for Tubular Atrophy. Please describe.

* must provide value

Expand

Interstitial infammation*:

(* Note: absent and minimal will be grouped together for analysis, so the distinction is not critical.)

* must provide value

- ◯ Absent/none (0%)
- ◯ Minimal (1-10%)
- ◯ Mild (10-25%)
- ◯ Mild-moderate (26-50%)
- ◯ Severe (>50% of cortex involved)
- ◯ Other
- ◯ Unknown

reset

You entered "Other" for interstitial infammation. Please describe.

* must provide value

Expand

Acute Tubular Injury (ATI) / Necrosis (ATN):*

(* Note: absent and minimal will be grouped together for analysis, so the distinction is not important.)

* must provide value

- ◯ Absent
- ◯ Minimal
- ◯ Mild (epithelial flattening, tubule dilation, nuclear dropout, loss of brush border)
- ◯ Mild-moderate (focal coagulative type necrosis)
- ◯ Severe (infarction)
- ◯ Other
- ◯ Unknown

reset

You entered "Other" for Acute Tubular Injury (ATI) / Necrosis (ATN). Please describe.

* must provide value

Expand

Biopsy Comments

Expand

Was another Biopsy Report found among the attachments?

* must provide value

- ◯ Yes
- ◯ No

reset

Biopsy 2 Information

Type of Biopsy Sample:

* must provide value

- ◯ Needle / Core
- ◯ Wedge
- ◯ Other
- ◯ Unknown

reset

Other Biopsy Sample Type:

* must provide value

Expand

Biopsy Preparation Method:

* must provide value

- ◯ Frozen section
- ◯ Fixed/permanent (parain-embedded)
- ◯ Other
- ◯ Unknown

reset

Other Biopsy Sample Preparation Method:

* must provide value

Expand

Number of Glomeruli Observed:

Number of Glomeruli Sclerosed:

% Glomerulosclerosis (Calculated Field): View equation

% Glomerulosclerosis (Direct Entry of Percentage)

Interstitial Fibrosis*:

(* Note: absent and minimal will be grouped together for analysis, so the distinction is not critical.)

* must provide value

- Absent/none (0%)
- Minimal (1-5%)
- Mild (6-25%)
- Mild-moderate (26-50%)
- Severe (>50% of cortex involved)
- Other
- Unknown

reset

You entered "Other" for Interstitial Fibrosis. Please describe.

* must provide value

Expand

Arterial intimal fibrosis (aka, arteriosclerosis or chronic vascular changes or vascular damage or vascular narrowing):*

(* Note: absent and minimal will be grouped together for analysis, so the distinction is not important.)

* must provide value

- Absent/none (0%)
- Minimal (0%)
- Mild (1-25%)
- Mild-moderate (26-50%)
- Severe (>50% vascular narrowing)
- Other
- Unknown

reset

You entered "Other" for Arterial intimal fibrosis (aka, arteriosclerosis or chronic vascular changes or vascular damage or vascular narrowing):. Please describe.

* must provide value

Expand

Arteriolar hyalinosis (strictly involves arterioles, the vessels without internal elastic lamina):*

(* Note: absent and minimal will be grouped together for analysis, so the distinction is not important.)

* must provide value

- Absent/none
- Minimal
- Mild (at least one arteriole)
- Mild-moderate (more than one arteriole)
- Severe (multiple arterioles affected, circumferential)
- Other
- Unknown

reset

You entered "Other" for Arteriolar hyalinosis (strictly involves arterioles, the vessels without internal elastic lamina). Please describe.

* must provide value

Expand

Tubular atrophy*:

(* Note: absent and minimal will be grouped together for analysis, so the distinction is not critical.)

* must provide value

- Absent/none (0%)
- Minimal (0%)
- Mild (1-25%)
- Mild-moderate (26-50%)
- Severe (>50% of cortical tubules involved)
- Other
- Unknown

reset

You entered "Other" for Tubular Atrophy. Please describe.

* must provide value

Expand

Interstitial infammation*:

(* Note: absent and minimal will be grouped together for analysis, so the distinction is not critical.)

* must provide value

- ○ Absent/none (0%)
- ○ Minimal (1-10%)
- ○ Mild (10-25%)
- ○ Mild-moderate (26-50%)
- ○ Severe (>50% of cortex involved)
- ○ Other
- ○ Unknown

reset

You entered "Other" for interstitial infammation. Please describe.

* must provide value

Expand

Acute Tubular Injury (ATI) / Necrosis (ATN):*

(* Note: absent and minimal will be grouped together for analysis, so the distinction is not important.)

* must provide value

- ○ Absent
- ○ Minimal
- ○ Mild (epithelial flattening, tubule dilation, nuclear dropout, loss of brush border)
- ○ Mild-moderate (focal coagulative type necrosis)
- ○ Severe (infarction)
- ○ Other
- ○ Unknown

reset

You entered "Other" for Acute Tubular Injury (ATI) / Necrosis (ATN). Please describe.

* must provide value

Expand

Biopsy Comments

Expand

Was another Biopsy Report found among the attachments?

* must provide value

- ○ Yes
- ○ No

reset

Biopsy 3 Information

Type of Biopsy Sample:

* must provide value

- ○ Needle / Core
- ○ Wedge
- ○ Other
- ○ Unknown

reset

Other Biopsy Sample Type:

* must provide value

Expand

Biopsy Preparation Method:

* must provide value

- ○ Frozen section
- ○ Fixed/permanent (parain-embedded)
- ○ Other
- ○ Unknown

reset

Other Biopsy Sample Preparation Method:

* must provide value

Expand

Number of Glomeruli Observed:

Number of Glomeruli Sclerosed:

% Glomerulosclerosis (Calculated Field): View equation

% Glomerulosclerosis (Direct Entry of Percentage)

Interstitial Fibrosis*:

(* Note: absent and minimal will be grouped together for analysis, so the distinction is not critical.)

* must provide value

- Absent/none (0%)
- Minimal (1-5%)
- Mild (6-25%)
- Mild-moderate (26-50%)
- Severe (>50% of cortex involved)
- Other
- Unknown

reset

You entered "Other" for Interstitial Fibrosis. Please describe.

* must provide value

Expand

Arterial intimal fibrosis (aka, arteriosclerosis or chronic vascular changes or vascular damage or vascular narrowing):*

(* Note: absent and minimal will be grouped together for analysis, so the distinction is not important.)

* must provide value

- Absent/none (0%)
- Minimal (0%)
- Mild (1-25%)
- Mild-moderate (26-50%)
- Severe (>50% vascular narrowing)
- Other
- Unknown

reset

You entered "Other" for Arterial intimal fibrosis (aka, arteriosclerosis or chronic vascular changes or vascular damage or vascular narrowing):. Please describe.

* must provide value

Expand

Arteriolar hyalinosis (strictly involves arterioles, the vessels without internal elastic lamina):*

(* Note: absent and minimal will be grouped together for analysis, so the distinction is not important.)

* must provide value

- Absent/none
- Minimal
- Mild (at least one arteriole)
- Mild-moderate (more than one arteriole)
- Severe (multiple arterioles affected, circumferential)
- Other
- Unknown

reset

You entered "Other" for Arteriolar hyalinosis (strictly involves arterioles, the vessels without internal elastic lamina). Please describe.

* must provide value

Expand

Tubular atrophy*:

(* Note: absent and minimal will be grouped together for analysis, so the distinction is not critical.)

* must provide value

- Absent/none (0%)
- Minimal (0%)
- Mild (1-25%)
- Mild-moderate (26-50%)
- Severe (>50% of cortical tubules involved)
- Other
- Unknown

reset

You entered "Other" for Tubular Atrophy. Please describe.

* must provide value

Expand

Interstitial infammation*:

(* Note: absent and minimal will be grouped together for analysis, so the distinction is not critical.)

* must provide value

- Absent/none (0%)
- Minimal (1-10%)
- Mild (10-25%)
- Mild-moderate (26-50%)
- Severe (>50% of cortex involved)
- Other
- Unknown

reset

You entered "Other" for interstitial infammation. Please describe.

* must provide value

Expand

Acute Tubular Injury (ATI) / Necrosis (ATN):*

(* Note: absent and minimal will be grouped together for analysis, so the distinction is not important.)

* must provide value

- Absent
- Minimal
- Mild (epithelial flattening, tubule dilation, nuclear dropout, loss of brush border)
- Mild-moderate (focal coagulative type necrosis)
- Severe (infarction)
- Other
- Unknown

reset

You entered "Other" for Acute Tubular Injury (ATI) / Necrosis (ATN). Please describe.

* must provide value

Expand

Biopsy Comments

Expand

Anatomy Information

Was an Anatomy Report found among the attachments?

* must provide value

- Yes
- No

reset

Kidney Length (cm)

Kidney Width (cm)

Surgical Damage:

* must provide value

- Yes
- No
- Other
- Unknown

reset

You entered "[Surgical_Damage]" for Surgical Damage. Please describe.

* must provide value

Expand

Anatomical Abnormalities:

* must provide value

- Yes
- No
- Other
- Unknown

reset

You entered "[Anatomical_Abnormalities]" for Anatomical Abnormalities. Please describe.

* must provide value

Expand

Number of Arteries:

* must provide value

Number of Veins:

* must provide value

Ureter:

* must provide value

- Single
- Double
- Other
- Unknown

reset

Ureter 1 Length (cm):

* must provide value

Ureter 2 Length (cm):

* must provide value

You entered "[Ureter]" for Ureter. Please describe.

* must provide value

Expand

Aortic Plaque:

* must provide value

- Yes
- No
- Other
- Unknown

reset

Aortic Plaque Type:

* must provide value

- Soft
- Hard
- Unknown

Aortic Plaque Severity

* must provide value

- Mild
- Moderate
- Severe
- Unknown

reset

You entered "[Aortic_Plaque]" for Aortic Plaque. Please describe.

* must provide value

Expand

Arterial Plaque

* must provide value

- Yes
- No
- Other
- Unknown

reset

Arterial Plaque Type

* must provide value

- Soft
- Hard
- Unknown

Arterial Plaque Severity

* must provide value

- Mild
- Moderate
- Severe
- Unknown

reset

You entered "[Arterial_Plaque]" for Arterial Plaque. Please describe.

* must provide value

Expand

Infarcted Areas

* must provide value

- Yes
- No
- Other
- Unknown

reset

Number of Infarcted Areas

* must provide value

You entered "[Infarcted_Areas]" for Infarcted Areas. Please describe.

* must provide value

Expand

Capsular Tears

* must provide value

- Yes
- No
- Other
- Unknown

reset

You entered "Other" for Capsular Tears. Please describe.

* must provide value

Expand

Subcapsular Hematomas

* must provide value

- Yes
- No
- Other
- Unknown

reset

You entered "Other" for Subcapsular Hematomas. Please describe.

* must provide value

Expand

Cysts/Discoloration

* must provide value

- Yes
- No
- Other
- Unknown

reset

Number of Cysts

* must provide value

Mean Cyst Size (cm^3)

You entered "Other" for Cysts/Discoloration. Please describe.

* must provide value

Expand

Fat Removed

* must provide value

- Yes
- No
- Other
- Unknown

reset

You entered "Other" for Fat Removed. Please describe.

* must provide value

Expand

Anatomy Comments

Expand
